# Sad1 Spatiotemporally Regulates Kinetochore Clustering To Ensure High-Fidelity Chromosome Segregation in the Human Fungal Pathogen *Cryptococcus neoformans*

**DOI:** 10.1128/mSphere.00190-18

**Published:** 2018-07-05

**Authors:** Vikas Yadav, Kaustuv Sanyal

**Affiliations:** a Molecular Biology and Genetics Unit, Jawaharlal Nehru Centre for Advanced Scientific Research, Bangalore, India; Yonsei University

**Keywords:** CENP-A, LINC complex, microtubule organizing center, mitotic spindle

## Abstract

Kinetochore clustering, frequently observed in yeasts, plays a key role in genome organization and chromosome segregation. In the absence of the metaphase plate arrangement, kinetochore clustering in yeast species is believed to facilitate timely kinetochore-microtubule interactions to achieve bivalent attachments of chromosomes during metaphase. The factors determining the dynamics of kinetochore clustering remain largely unknown. We previously reported that kinetochores oscillate between an unclustered and a clustered state during the mitotic cell cycle in the basidiomycetous yeast Cryptococcus neoformans. Based on tubulin localization patterns, while kinetochore clustering appears to be microtubule dependent, an indirect interaction of microtubules with kinetochores is expected in C. neoformans. In this study, we sought to examine possible roles of the SUN-KASH protein complex, known to form a bridge across the nuclear envelope, in regulating kinetochore clustering in C. neoformans. We show that the SUN domain protein Sad1 localizes close to kinetochores in interphase as well as in mitotic cells. Sad1 is nonessential for viability in C. neoformans but is required for proper growth and high-fidelity chromosome segregation. Further, we demonstrate that the onset of kinetochore clustering is significantly delayed in cells lacking Sad1 compared to wild-type cells. Taken together, this study identifies a novel role of the SUN domain protein Sad1 in spatiotemporal regulation of kinetochore clustering during the mitotic cell cycle in C. neoformans.

**IMPORTANCE** The linker of nucleoskeleton and cytoskeleton (LINC) complex is present in fungi, animals, and plants. It performs diverse functions in animals, and its role(s) have recently been explored in plants. In ascomycetous yeast species, the role of the LINC complex in spindle pole body function and telomere clustering during meiosis has been determined. However, nothing is known about the LINC complex in the fungal phylum of Basidiomycota. In this study, we identified the role of the LINC complex in kinetochore dynamics as well as in nuclear migration in a basidiomycetous yeast, Cryptococcus neoformans, a human pathogen. Unlike most other yeast species, kinetochores remain unclustered during interphase but gradually cluster during mitosis in C. neoformans. We report that the LINC complex is required for timely onset of kinetochore clustering and high-fidelity chromosome segregation in C. neoformans. Thus, our study identifies a novel factor required for kinetochore clustering during mitosis in yeast species.

## Abstract (precis)

The linker of nucleoskeleton and cytoskeleton (LINC) complex is present in fungi, animals, and plants. It performs diverse functions in animals, and its role(s) have recently been explored in plants. In ascomycetous yeast species, the role of the LINC complex in spindle pole body function and telomere clustering during meiosis has been determined. However, nothing is known about the LINC complex in the fungal phylum of Basidiomycota. In this study, we identified the role of the LINC complex in kinetochore dynamics as well as in nuclear migration in a basidiomycetous yeast, Cryptococcus neoformans, a human pathogen. Unlike most other yeast species, kinetochores remain unclustered during interphase but gradually cluster during mitosis in C. neoformans. We report that the LINC complex is required for timely onset of kinetochore clustering and high-fidelity chromosome segregation in C. neoformans. Thus, our study identifies a novel factor required for kinetochore clustering during mitosis in yeast species.

## INTRODUCTION

Centromeres/kinetochores are clustered at the nuclear periphery in most studied yeast species (1–4). Kinetochore clustering helps in the organization of yeast chromosomes in the Rabl configuration so that chromosome arms lie freely in the nucleoplasm (5). In budding yeast species Saccharomyces cerevisiae and Candida albicans, kinetochores are clustered throughout the cell cycle (1–3, 6). However, in the fission yeast Schizosaccharomyces pombe, kinetochores are clustered in interphase but uncluster during mitosis (4). Despite this difference, kinetochore clustering is required for proper kinetochore-microtubule attachment during the onset of mitosis in these yeast species (6–8). Kinetochore clustering also facilitates compartmentalization of chromatin into multiple functional domains required for processes such as DNA repair and a high level of transcription in the interphase nucleus (5, 9, 10). Beyond yeast species, kinetochore clustering is also observed in the mitotic cells of Drosophila melanogaster, where kinetochores are present close to the nucleolus and play a significant role in heterochromatin organization (11, 12). During early stages of meiosis, the kinetochore cluster also facilitates proper synapse formation in *Drosophila* (13, 14). On the other hand, kinetochores do not cluster at any stage of the cell cycle in most metazoans, where the formation of the metaphase plate aligns all chromosomes on a single plane.

A series of observations revealed a diverse group of proteins that contribute to the process of kinetochore clustering to ensure proper chromosome segregation. In S. cerevisiae, clustered kinetochores are always associated with the spindle pole bodies (SPBs) directly through microtubules (15). An outer kinetochore protein, Slk19, is also required in addition to microtubules in this organism (8). In S. pombe, kinetochores are held close to the SPBs by an indirect link involving proteins such as Sad1, Ima1, and Csi1 (7, 16, 17). Disruption of microtubules alone does not have any effect on kinetochore clustering in this organism (18). A nucleoplasmin homolog, NLP, is required for maintaining the kinetochore cluster close to the nucleolus in *Drosophila* (11).

The linker of nucleoskeleton and cytoskeleton (LINC) complex forms a bridge across the nuclear envelope (NE) in most eukaryotes (19, 20). The LINC complex consists of KASH (Klarsicht, ANC-1, and syne homology) domain proteins present in the outer nuclear membrane and SUN (Sad1 and UNC-84) domain proteins in the inner nuclear membrane. The SUN domain is a motif that is highly conserved across evolution, whereas the KASH domain is comprised of a highly variable stretch of 50 to 60 amino acids that typically ends with “PPPX” (21–23). The KASH and SUN domains present at the C terminal of corresponding proteins interact with each other in the perinuclear space to establish the LINC complex. The N terminal of KASH proteins extends into the cytoplasm and interacts with cytoskeletal elements, whereas the N terminal of SUN proteins interacts with lamins and chromatin-associated proteins in the nucleoplasm. Due to its ability to transfer mechanical force across the NE, the LINC complex plays essential roles in a variety of cellular processes, including chromatin organization, nuclear division, and signal transduction (24, 25). SUN-KASH proteins are closely associated with the SPBs in yeast species. In S. pombe, these proteins tether the kinetochores to SPBs during mitosis to facilitate kinetochore clustering, and they tether telomeres to SPBs during meiosis to ensure telomere clustering (26, 27). The SUN protein Mps3 interacts with a substructure called the half-bridge in S. cerevisiae. The interaction between Mps3 and the half-bridge is essential for proper functioning as well as duplication of SPBs (28, 29). The roles of the LINC complex in nuclear dynamics as well as nuclear structure maintenance are well studied in C. elegans (30, 31). SUN-KASH proteins are also known to play a critical role in meiotic chromosome pairing and synapsis formation in both yeast and mammals (32–34).

In this study, we examined the role of Sad1, a SUN domain protein, in kinetochore clustering in a basidiomycete yeast, Cryptococcus neoformans. While the role of Sad1 in kinetochore dynamics has been recently implicated in S. pombe (16), an ascomycete, its role in basidiomycetes yeast species is unknown. Moreover, the dynamics of kinetochore clustering is different in S. pombe and C. neoformans. Kinetochores in C. neoformans are unclustered during interphase but begin to cluster as a cell enters mitosis (35). The microtubules were found to be essential for kinetochore clustering in this organism. However, an apparent absence of nuclear microtubules during interphase hinted toward an indirect interaction between the kinetochore and microtubules. Here, we show that Sad1 colocalizes with CENP-A, which forms centromeric chromatin and marks the kinetochores, suggesting their close association at all stages of the cell cycle in C. neoformans. A population of *sad1* null mutant cells exhibited gross chromosome segregation defects and a significant delay in kinetochore clustering compared to wild-type cells. Overall, these results establish a novel function of the SUN domain protein in regulating spatiotemporal dynamics of kinetochore clustering in a basidiomycete yeast, C. neoformans.

## RESULTS AND DISCUSSION

### MTOCs localize close to the kinetochore.

**FIG 1:**
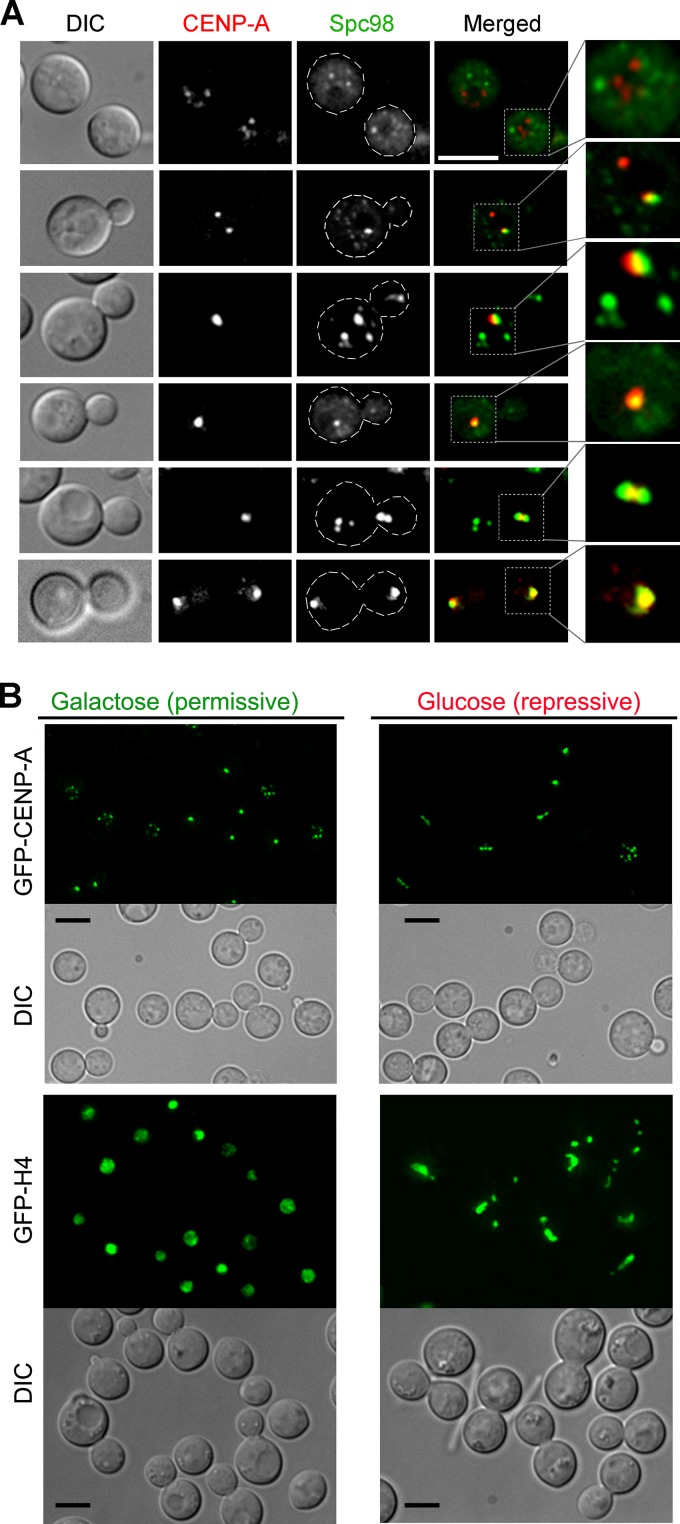
Dynamic colocalization of MTOCs and the kinetochore during the cell cycle in C. neoformans. (A) Dynamics of colocalization of the kinetochore (mCherry–CENP-A) and MTOCs (Spc98-GFP) at different stages of the cell cycle in C. neoformans. (B) *GAL7p*-Spc98 conditional mutant was grown in both permissive and repressive media for 12 h. The cells were harvested and studied for the status of kinetochore (marked by GFP–CENP-A) and chromatin (marked by GFP-H4) localization. The cells depleted of Spc98 showed an aberrant kinetochore localization pattern and massive chromosome segregation errors. On the other hand, the cells overexpressing Spc98 behaved in the same fashion as wild-type cells and did not show any difference in kinetochore or chromatin localization. Bars, 5 µm.

Kinetochores in S. pombe and S. cerevisiae are clustered and localize close to SPBs that are embedded in the nuclear membrane (36). In contrast, kinetochores in C. neoformans are unclustered during interphase (35). Moreover, a previous report in C. neoformans suggested that SPBs are not embedded in the NE but are localized to the cytoplasm, close to the outer nuclear membrane (37). We localized Spc98 labeled with green fluorescent protein (Spc98-GFP), a subunit of microtubule organizing centers (MTOCs) which coalesce to form SPBs, and mCherry-CENP-A, which marks the kinetochore, in C. neoformans in order to understand the association of MTOCs/SPBs with the kinetochore. In unbudded interphase cells, MTOC puncta seem to localize in regions mostly excluded from the kinetochore signals, indicating that MTOCs are scattered throughout the cytoplasm (Fig. 1A). These localization patterns of MTOCs are similar to MTOC dynamics observed in another basidiomycete, Ustilago maydis (38). However, a fraction of Spc98 puncta in C. neoformans localized close to the CENP-A dot-like signals in interphase cells, indicating dynamic and transient colocalization dynamics of kinetochores and MTOCs (Fig. 1A). In addition, such observed partial colocalization can be an artifact of the image projection algorithm. A lack of constitutive colocalization between the SPBs and kinetochores further suggested that they may not interact directly with each other. As the cell cycle progressed, the Spc98-GFP signals gradually clustered, probably at the SPB, and localized close to the clustered kinetochores, followed by their transition to the daughter cell. Subsequently, signals representing either clustered MTOCs or clustered kinetochores segregated into two halves during mitosis, one of which in each case then moved back to the mother cell while the other remained in the daughter cell. We previously reported the dynamics of microtubules and kinetochores (35), which are similar to the dynamics observed between MTOCs and kinetochores. These results indicate that MTOCs or microtubules do not interact with kinetochores until the onset of mitosis, when kinetochores begin to cluster. Indeed, a direct interaction between microtubules and kinetochores is least expected during interphase due to the presence of the NE as a barrier. In contrast, our previous results suggested that microtubules are required for kinetochore clustering, which takes place before a kinetochore-microtubule attachment is established (35). In addition, disruption of MTOCs by repressing Spc98 using the *GAL7* promoter resulted in a linear array of distinct GFP–CENP-A dots in most large budded cells instead of a single clustered dot as observed in wild-type large budded cells (Fig. 1B). However, the kinetochore clustering dynamics when Spc98 was overexpressed was identical to that in wild-type cells, indicating that the elevated levels of Spc98-GFP did not interfere with this process. The conditional mutant cells in the absence of Spc98 (grown in glucose) also exhibited massive nuclear segregation defects as opposed to proper nuclear segregation observed in cells grown in galactose (Fig. 1B). These results confirmed that microtubules play a major role in kinetochore clustering and nuclear division. The presence of individual unclustered GFP–CENP-A dot-like signals in the absence of MTOCs suggested that the kinetochore clustering is affected in the absence of microtubules. Thus, we hypothesized that MTOCs/microtubules and kinetochores interact with each other through an indirect link probably involving an active component of the NE.

### Sad1, a SUN domain protein, localizes close to kinetochores in C. neoformans.

**FIG 2:**
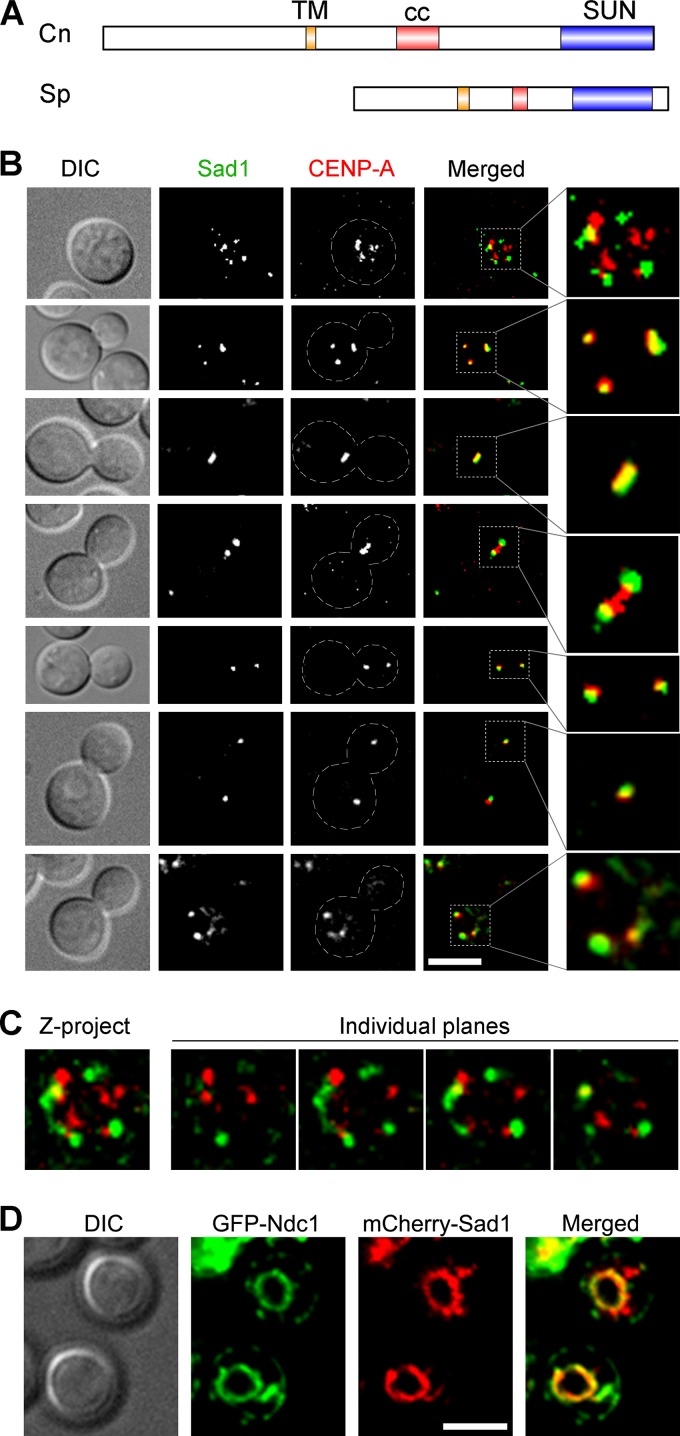
Sad1 localizes close to the kinetochore throughout the cell cycle in C. neoformans. (A) The domain architecture of C. neoformans Sad1 (CnSad1) and its comparison with S. pombe Sad1 (SpSad1). TM, transmembrane domain; cc, coiled-coil region; SUN, SUN domain. (B) Colocalization of C. neoformans Sad1-GFP with mCherry–CENP-A, a kinetochore marker, reveals a close association between two proteins throughout the cell cycle. Bar, 5 µm. (C) Colocalization analysis of the unbudded cell shown in panel B revealed that a fraction of kinetochore signals (red) and Sad1 (green) colocalized (yellow) during interphase. The colocalization was observed even in individual plane images, indicating a direct interaction between the kinetochore and Sad1. Some of the CENP-A signals that did not colocalize with Sad1 might have been a result of microscopy imaging limitations. (D) Snapshots depicting the localization of the NE (GFP-Ndc1), and mCherry-Sad1 in unbudded cells. mCherry-Sad1 was expressed using the *GAL7* promoter and localized along the NE. Bar, 5 µm.

Two protein complexes, the nuclear pore complex (NPC) and the LINC complex, are well known to provide a link between the cytoplasm and the nucleoplasm. We previously reported that NPCs do not colocalize with the kinetochore but disappear upon the onset of mitosis in C. neoformans (35). The LINC complex, comprised of SUN and KASH domain proteins, remains unidentified in C. neoformans. Based on an *in silico* search using the fission yeast SUN domain protein as a query in a BLAST analysis, we identified a SUN domain-containing protein, Sad1 in C. neoformans (Fig. 2A). *In silico* domain prediction for the Sad1 protein sequence in C. neoformans revealed the presence of a coiled-coil region and a transmembrane domain along with the signature SUN domain found in Sad1 in S. pombe (SpSad1). No KASH domain protein, however, could be identified in our analysis, probably due to an absence of a conserved sequence motif. Next, we expressed GFP-tagged Sad1 to study its relative localization with the kinetochore protein mCherry–CENP-A. This study revealed a dynamic association between the two proteins (Fig. 2B and C). During interphase, both proteins localized as multiple puncta in a common area that appeared circular in a single focal plane and most likely represents the NE (35). The Sad1 localization at the nuclear periphery was supported by colocalization of overexpressed mCherry-Sad1 with Ndc1, an NE marker (Fig. 2D). While in all unbudded cells examined a partial colocalization was observed, the two signals were mostly nonoverlapping. Unlike Spc98-GFP, Sad1-GFP dot-like signals were restricted only to the NE without any cytoplasmic localization. Strikingly, in budded cells that initiated clustering of kinetochores, more extensive colocalization of Sad1 and CENP-A was observed than in interphase cells (Fig. 2B, compare images in first and the second row). In cells where clustering of kinetochores was completed, the two proteins completely colocalized, and this colocalization persisted until anaphase. In early anaphase (fourth row), Sad1 appeared as two dots on two sides of a rod-like signal of CENP-A. Later in anaphase, Sad1 and CENP-A signals nearly completely overlapped again, forming two dots. In telophase, Sad1 and CENP-A signals showed less overlapping, similar to interphase cells. Taken together, these data suggest that prior to kinetochore clustering, most kinetochores do not interact with Sad1. Upon clustering initiation, Sad1 associates with kinetochores, either directly or indirectly, and this association persists until telophase. Based on these observations, we envisioned a possible mechanism responsible for kinetochore clustering where upon initiation of clustering, kinetochores connect to microtubules/MTOCs via Sad1. Whether this link continues to act even after complete clustering is not clear, because microtubules too can completely attach to assembled kinetochores directly during mitosis. The LINC connection between kinetochores and microtubules during interphase would also explain the effect of microtubule disruption on the kinetochore dynamics in C. neoformans.

### Sad1 is required for timely clustering of kinetochores.

**FIG 3:**
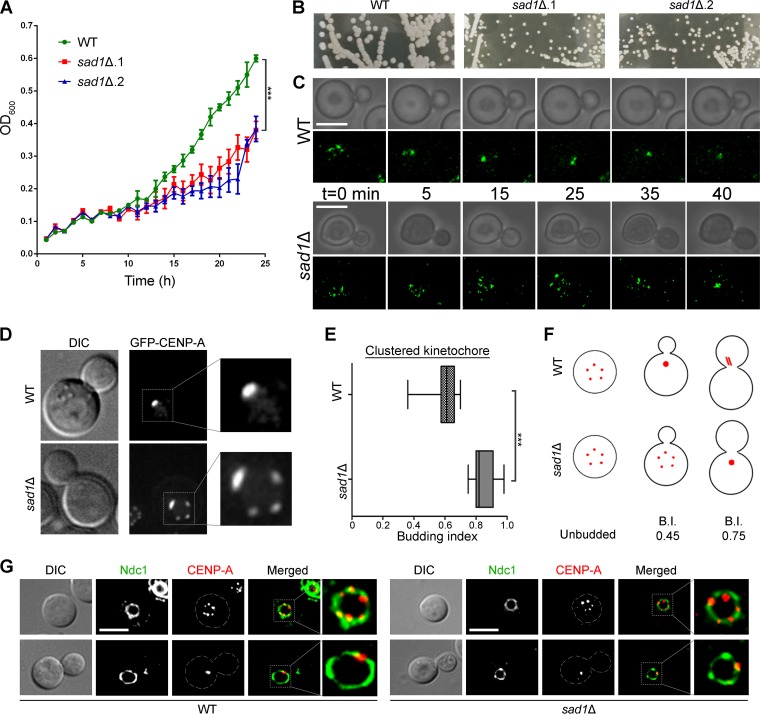
Kinetochore clustering is delayed in the *sad1* null mutant. (A) A graph showing the growth rates of wild-type and *sad1*Δ mutant cells (*P* < 0.0001). (B) Plate images showing the colonies formed by both the wild type and the *sad1*Δ mutants on the YPD plates after 4 days. The images shown were captured at the same magnification. (C) Time-lapse imaging showing kinetochore clustering in both wild-type and *sad1*Δ mutant cells (*n* = 5). Bars, 5 µm. (D) Snapshots depicting the status of the kinetochore clustering in the mutant and wild-type cells of a similar budding index. (E) The kinetochore clustering status was correlated with the budding index (BI) and was plotted for cells with the clustered kinetochores (*n* = 50). As shown, kinetochore clustering was delayed in the mutant and took place only when cells attained a BI of 0.7, while it occurred at a BI of 0.4 in the wild-type cells (*P* < 0.0001). (F) A cartoon depicting kinetochore clustering dynamics in both the wild type and the *sad1*Δ mutant. (G) Localization of the kinetochore (mCherry–CENP-A) with respect to the nuclear envelope (GFP-Ndc1) does not change in *sad1* null cells compared to the wild-type cells, in both interphase and mitotic cells.

The SUN domain proteins are essential for viability in most organisms studied, including the fission yeast S. pombe and the budding yeast S. cerevisiae (28, 29). The essentiality of these proteins for the viability of cells make it difficult to study the direct role of a SUN domain protein in the kinetochore dynamics in any organism. Unlike S. pombe and S. cerevisiae, the *sad1* null mutant was found to be viable in C. neoformans. However, the null mutant cells exhibited severe growth defects (Fig. 3A and B). We expressed GFP–CENP-A in* sad1* null cells and performed real-time live cell imaging. First, we examined small budded cells (budding index of 0.2) of the wild type and the *sad1* null mutant. The wild-type cells displayed complete clustering of kinetochores in ≤25 min, whereas the null mutant cells required ≥40 min for the same (Fig. 3C). We also correlated kinetochore clustering time with the budding index. Previously, we demonstrated that CENP-A signals cluster in C. neoformans by the time the cell attains the budding index of 0.4 (35). We employed these correlative parameters to quantify the extent of delay in kinetochore clustering in the absence of Sad1. We measured the budding index of cells harboring clustered CENP-A signals as a single punctum in both wild-type and mutant cells. As expected, kinetochores were clustered in wild-type cells as soon as the budding index of 0.4 was attained. The *sad1* null cells, on the other hand, could cluster kinetochores only when the budding index was ≥0.7 (Fig. 3D to F). These results confirm that Sad1 plays a significant role in the timely onset of kinetochore clustering in C. neoformans. However, the peripheral localization of kinetochores is not perturbed in *sad1* null cells, indicating that other protein complexes might also play a role in tethering the kinetochores to the NE (Fig. 3G). Proteins like Ima1, Lem2, and Csi1 have been described to play such tethering roles in S. cerevisiae and S. pombe (7, 16, 17).

### Sad1 is required for proper spindle localization to ensure equal nuclear division during mitosis.

**FIG 4:**
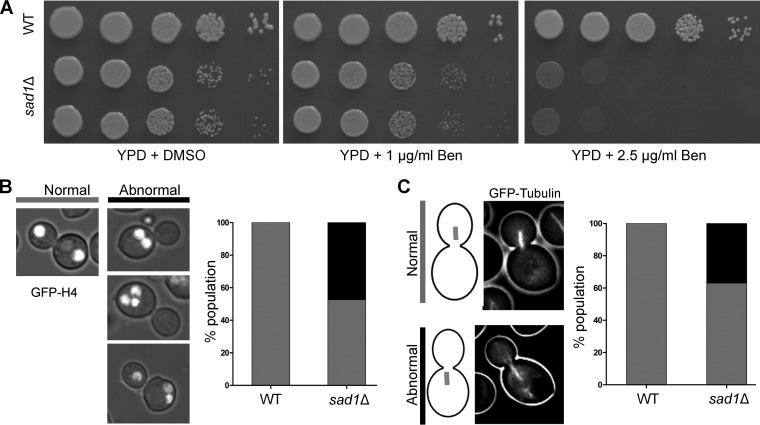
Sad1 is required for proper chromosome segregation. (A) Plate images displaying the sensitivity of the *sad1*Δ mutant to a microtubule-depolymerizing drug, benomyl (Ben), compared to the wild-type cells. The mutant grows slower on the control (dimethyl sulfoxide [DMSO]) plate, as shown in Fig. 3A. (B) A graph showing the status of chromosome segregation (marked by GFP- histone H4) in the wild-type and the *sad1*Δ mutant large budded cells (budding index, ≥0.75; *n* = 50). (C) A graph depicting localization patterns of the mitotic spindle in the *sad1*Δ mutant compared to that in the wild-type large budded cells (*n* = 30).

Defects in kinetochore clustering lead to abnormal chromosome segregation in many yeast species (7, 8). The *sad1* null cells suffer a significant delay in kinetochore clustering in C. neoformans. Absence of Sad1 can also lead to a significant reduction in the pulling force that is exerted on chromatin during mitosis for proper nuclear dynamics. The mutant cells exhibited a higher sensitivity to the microtubule-depolymerizing drug benomyl than did the wild type (Fig. 4A), suggesting the role of Sad1 in a kinetochore-microtubule-mediated process of chromosome segregation. To assess the effect of loss of Sad1 on chromosome segregation, a *sad1* null strain was generated where the nucleus was marked with GFP-tagged histone H4. Analysis of GFP-histone H4 signals in the mutant revealed a high rate (~50%) of chromosome missegregation compared to that in the wild-type cells (Fig. 4B). The most common phenotype observed in the mutant was the presence of two nuclear masses in a single cell. In some cells, three nuclei were observed, which can arise if there are lagging chromosomes after the first nuclear division. Alternatively, the three nuclei per cell may arise when one of the two nuclei undergoes a second round of division. This second round of nuclear division can lead to normal chromatin movement, resulting in two nuclei in the mother cell and one nucleus in the daughter cell. On the other hand, abnormal migration in the second round of division can also give rise to three nuclei in the mother cell itself. Overall, this result confirms that the proper nuclear dynamics are altered in the mutant cells, leading to defective chromosome segregation. We previously demonstrated that the nuclear dynamics during mitosis in C. neoformans is dependent on the number and integrity of cytoplasmic microtubules (35, 39). We observed that the *sad1* null mutant cells do not show any significant difference in cytoplasmic microtubules during interphase. However, examination of the mitotic spindle in *sad1* null cells revealed a large number of mutant cells (~40%) with the mitotic spindle mispositioned in the mother cell (Fig. 4C), in contrast to the wild-type cells in which the mitotic spindle always formed in the daughter cell. Strikingly, the fractions of cell populations having the nuclear segregation defects and mitotic spindle defects are similar. These defects could also account for the slow growth observed in the *sad1* mutant (Fig. 3A and 4A). Based on these results, we conclude that aberrant chromosome segregation in the *sad1* null cells arises due to irregular premitotic nuclear migration. A similar phenotype was observed in the *dynein* mutant of Ustilago maydis (40). The defect was attributed to a lack of force on chromatin that is exerted by the microtubules through dynein, a motor protein. SUN-KASH proteins are known to interact with microtubules through various motors, including dynein (41). Thus, it is possible that in the absence of Sad1, chromatin fails to experience enough force required for its movement to the daughter cell—the proper site for nuclear division in this organism. The lack of movement could eventually lead to the division of the nucleus in the mother cell, giving rise to two segregated nuclear masses in the same mother cell and none in the daughter.

**FIG 5:**
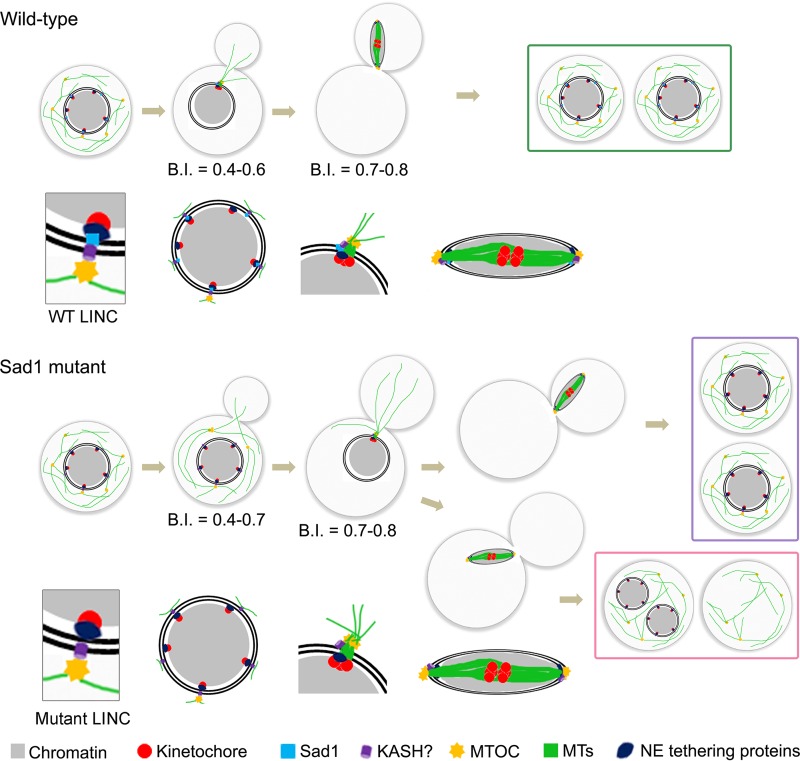
A model describing the role of Sad1 protein in kinetochore clustering in C. neoformans. In a wild-type cell, the timely onset of kinetochore clustering allows proper kinetochore-microtubule attachment. The chromatin moves to the daughter cell in a microtubule-dependent manner followed by segregation of the sister chromatids. In the absence of Sad1, the kinetochore clustering is delayed, perturbing the critical timing of kinetochore-microtubule attachment. This leads to abnormal nuclear dynamics and mislocalization of the mitotic spindle, eventually resulting in chromosome missegregation in a population of cells. Remaining cells divide normally, giving rise to two separate nuclei, one each in the mother cell and the daughter cell. A zoomed view of the LINC complex localization at each cell cycle stage is shown for both wild-type and Sad1 mutant cells. LINC, linker of nucleoskeleton and cytoskeleton; MTs, microtubules; MTOC, microtubule-organizing center; NE, nuclear envelope.

Kinetochore clustering is an essential yet relatively poorly studied phenomenon in yeast species. A series of studies identified some factors that are required for normal kinetochore clustering in various yeast species. In this study, we identified a SUN domain protein, Sad1, and its role in kinetochore clustering in a basidiomycete yeast, C. neoformans. Sad1 localizes close to kinetochores and is required for the timely onset of kinetochore clustering. We also found that a delay in kinetochore clustering results in defective mitotic spindle localization and chromosome missegregation in this organism. Based on these results, we propose that interaction between Sad1 and chromatin is critical for the spatiotemporal dynamics of kinetochore clustering that ensures proper nuclear dynamics for high-fidelity chromosome segregation in C. neoformans (Fig. 5). It is important to note that the nuclear division in C. neoformans takes place after the entire nuclear mass is transferred to the daughter cell through a biased, directed dynamics of microtubules (39). In a wild-type cell, microtubules transfer their forces to chromatin through the centromere-kinetochore complex via Sad1, a part of the SUN-KASH bridge or a similar complex that may be present in C. neoformans (Fig. 5). In the absence of Sad1, the connection between chromatin and microtubules is lost, and the pulling force is restricted to the NE instead of reaching the chromatin mass. This could give rise to various unusual scenarios: (i) the NE along with chromatin migrates to the daughter cell, (ii) the NE ruptures due to an excess force, or (iii) the nucleus is unable to move to the daughter cell due to lack of sufficient magnitude of force required. The phenotypes displayed by the *sad1* null cells revealed that approximately half of the mutant population harbors defects in nuclear migration and the spindle localization.

A recent report demonstrated a direct role of Sad1 in establishment and maintenance of clustered kinetochores in S. pombe (16). In the absence of this interaction, cells formed a defective spindle which was rescued when the SPB-kinetochore connection was restored artificially. The timely onset of kinetochore clustering in C. neoformans also requires the presence of Sad1. However, in contrast to S. pombe, the mitotic spindle formation is not perturbed in C. neoformans* sad1* mutant cells; rather, the location of the spindle is found to be altered in a large population among the mutant cells. Further, SpSad1 was proposed to interact with the outer kinetochore complex earlier (7, 42). In C. neoformans, the outer kinetochore proteins are not loaded to the kinetochore in interphase, indicating that Sad1 may interact with some inner kinetochore proteins (35). Hence, though the process of kinetochore clustering is affected in both these organisms due to lack of Sad1, the underlying mechanisms differ. These variations may be attributed to fundamental differences in the process of mitotic division in these two organisms.

Overall, we describe here a novel role played by a SUN domain protein in the kinetochore clustering dynamics. The roles of the LINC complex are well-established in animals (19, 24). Recently, a number of studies identified diverse functions for SUN-KASH proteins in plants (22, 23). In fungi, the role of SUN-KASH proteins is understood only for a few species belonging to Ascomycota, and their role has been explored primarily in association with SPBs (16, 29). Our study describes a component of the LINC complex in a Basidiomycota and a novel role of this protein in the kinetochore dynamics and high-fidelity chromosome segregation. The role of Sad1 in basidiomycetes differs from that in ascomycetes with respect to its association with SPBs in premitotic cells. Loss of Sad1 function in C. neoformans leads to slower growth, but the protein is not essential for viability, indicating that other compensating mechanisms may exist in this organism. Investigating the role of proteins like Ima1, lem2, and KASH proteins will provide further insights into the kinetochore clustering mechanism in this organism. Also, the role of Sad1 and other proteins during meiosis can be studied in C. neoformans because Sad1 interacts with telomeres in other yeast species during meiosis and telomere dynamics have not been studied in basidiomycetes.

## MATERIALS AND METHODS

### Strains and media.

The strains and plasmids used in this study are listed in Table 1. C. neoformans strains were grown in YPD (1% yeast extract, 2% peptone, and 2% dextrose) medium at 30°C with shaking at 180 rpm, unless otherwise specified. C. neoformans cells were transformed using biolistics as described previously (43). The transformants were selected on YPD or YPG (1% yeast extract, 2% peptone, and 2% galactose) containing either 200 µg/ml of G-418 (catalog number A1720; Sigma), 100 µg/ml of nourseothricin (product 5.0; Werner BioAgents), or 200 µg/ml of hygromycin (catalog number 10687-010; Invitrogen). The transformants were screened for correct integration via PCR and/or Western blotting.

**TABLE 1:** Strains and plasmids used in this study

Strain or plasmid	Strain genotype or plasmid construction	Source
Strains		
CNVY106	α H99::*GFP-tubulin-NAT* (pLKB35)	This study
CNVY108	α H99::*GFP-H4-NAT* (pVY3)	35
CNVY111	**a** KN99::*mCherry-CENP-A-NEO* (pLKB74) *GFP-NDC1-NAT* (pVY4)	35
CNVY138	**a** KN99::*mCherry-CENP-A-NEO* (pLKB74) *SAD1*::*SAD1-GFP-NAT*	This study
CNVY156	α H99::*CENP-Ap-GFP-CENP-A-NAT* (pVY22)	This study
CNVY177	α* SPC98p*::*GAL7p-SPC98-HYG SPC98*::*SPC98-GFP-NAT* H99*::mCherry-CENP-A-NEO* (pLKB74)	This study
CNVY182	α H99 *SAD1p*::*GAL7p-mCherry-SAD1-HYG GFP-NDC1-NAT* (pVY4)	This study
CNVY191	α* SAD1*::*sad1-NEO*	This study
CNVY193	**a** KN99::*mCherry-CENP-A-HYG* (pLKB75) *GFP-NDC1-NAT* (pVY4) *SAD1*::*sad1-NEO*	This study
CNVY194	α H99::*GFP-tubulin-NAT* (pLKB35) *SAD1::sad1-NEO*	This study
CNVY200	**a** KN99::*CENP-Ap-GFP-CENP-A-NAT* (pVY8) *SAD1*::*sad1-NEO*	This study
CNVY210	α H99::*GFP-H4-NAT* (pVY3) *SAD1*::*sad1-NEO*	This study
Plasmids		
pLKB35	pCN19 + α-tubulin (BamHI-BamHI)	35
pLKB74	pXLI + *CENP-Ap*-mCherry-CENP-A (NEO)	35
pLKB75	pXLI + *CENP-Ap*-mCherry-CENP-A (HYG)	35
pVY1	pCN19 + CENP-A (BamHI-BamHI)	35
pVY3	pCN19 + H4 (BamHI-SpeI)	35
pVY4	pCN19 + NDC1 (BamHI − SpeI)	35
pVY8	GFP-CENP-A-NAT from pVY1 into pBSII KS using SacI-ApaI	This study
pVY22	*CENP-Ap* replaced H3p in pVY8	This study

### Construction of fluorescently tagged proteins.

To tag the desired protein with GFP or mCherry at the C terminus, the overlap PCR strategy was used as described previously (35, 44). Specifically, *SAD1* (CNAG_03781) and *SPC98* (CNAG_01566) were tagged with GFP by using the constructs generated by the overlap PCR strategy. For this purpose, 1 kb each of the 3′ part of the gene (without the stop codon) and 3′ untranslated region (UTR) after the stop codon was amplified from the H99 genome. A GFP-NAT fragment (approximately 3 kb) was amplified from pCN19 (44), and all three fragments were fused by overlap PCR, generating the cassettes. The resulting cassettes were transformed in H99 using biolistics, and PCRs were performed to confirm the correct genomic integrations. To express GFP–CENP-A from the native promoter of CENP-A, a SacI-ApaI fragment containing sequence from pVY1 (35) was subcloned into pBlueScriptII KS(−) to generate pVY8. The *CENP-A* promoter (702 bp of upstream sequence of the *CENP-A* open reading frame [ORF]) was then amplified and cloned as the SacI-NcoI fragment into respective sites of pVY8 to generate pVY22. The plasmid was then transformed in H99 to express GFP-tagged CENP-A from its native promoter.

The expression level of Spc98 was found to be very low, and signals could not be detected when the fusion protein was expressed from its native promoter. To enhance the expression, the Spc98-GFP fusion protein was expressed using the *GAL7* promoter (45). The overlap PCR strategy was used to generate a construct to replace the native promoter of *SPC98* with the *GAL7* promoter. An approximately 1-kb region from the 5′ UTR as an upstream sequence (US) and a region of the similar length of the ORF, including start codon ATG, as a downstream sequence (DS), were amplified from the H99 genome. The middle fragment of approximately 2 kb, containing the hygromycin resistance gene and the *GAL7* promoter (*GAL7p*) region (~2 kb), was amplified from a plasmid harboring hygromycin and *GAL7p* cloned together. Three products were purified and used for overlap PCR to give rise to the full-length cassette. The cassette was transformed into a strain where *SPC98* was already tagged with GFP, and transformants were screened by PCR.

The *SAD1* gene deletion cassette was also generated by the overlap PCR strategy. For this purpose, a 1-kb region upstream of the start codon and 1-kb region downstream beyond the stop codon were amplified separately. The third fragment of 2 kb containing the neomycin gene was amplified from pLK25 (44). The three parts were purified and fused together to generate the final deletion construct of 3.8 kb. The full-length construct was transformed into C. neoformans strains, and correct integrants were confirmed by PCR.

### Growth curve assay.

Cells of C. neoformans wild type and *sad1* null mutants grown overnight were diluted into fresh YPD medium to obtain an optical density at 600 nm (OD_600_) of 0.05. The diluted cultures were aliquoted in a 96-well plate with 100 µl culture in a single well. Each strain was aliquoted in triplicate and grown for 24 h at 30°C with continuous shaking at 300 rpm. The OD_600_ of the wells was measured using a Varioskan Flash spectral scanning multimode reader (Thermo Fisher) at 1-h intervals. The final OD values were calculated by subtracting blank (only YPD) control OD values, and the growth curve was plotted using GraphPad Prism.

### Microscopy.

For microscopy, cells were grown in YPD broth with shaking at 180 rpm for 14 to 16 h and pelleted at 4,000 rpm. Cells were then washed once with distilled water and finally resuspended in distilled water. Cells were observed, and images were captured at 100× using a confocal laser scanning microscope, LSM 510 META or LSM880 (Carl Zeiss, Inc.) or the DeltaVision system (Applied Precision). For live cell imaging, an overnight YPD culture was diluted in the fresh synthetic complete growth medium and grown for 3 h. Next, ~0.5 µl of cell suspension was placed on a slide containing a thin patch of 2% agarose with complete medium, and a coverslip was placed on top of it. Images were captured at 100× using a confocal laser scanning microscope LSM880 (Carl Zeiss, Inc.). The image processing was done using either Zeiss image processing software LSM5 Image Examiner, ImageJ, or Adobe Photoshop (Adobe Systems).

### Budding index calculations.

Budding index was calculated for 50 cells each for the wild-type and the *sad1* null mutant strains. The diameters of the mother cell and the daughter cell were measured by using either the Image Pro-plus software or LSM software. The diameter value of the daughter cell was then divided by that of the mother cell to obtain the ratio, which was defined as the budding index.
